# Supplementary figures and images for: A bone-specific adipogenesis pathway in fat-free mice defines key origins and adaptations of bone marrow adipocytes with age and disease
Source: eLife. 2021 Aug 11;10:e66275. doi: 10.7554/eLife.66275 (PMC8412938; doi:10.7554/eLife.66275)

Figure 2-Source Data 2. Source Data for Panel A.

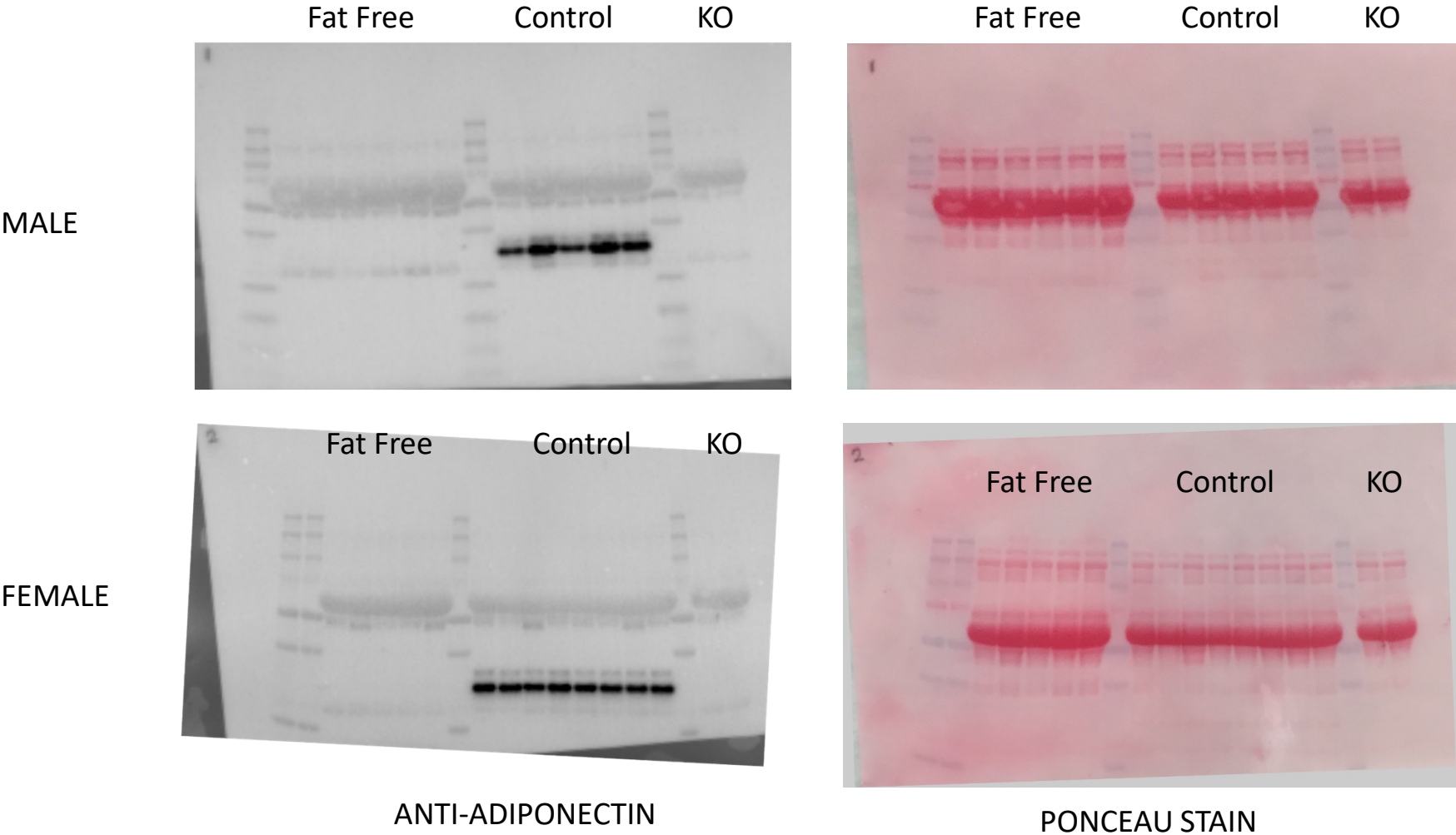

Supplement: Figure 2—source data 2. [file elife-66275-fig2-data2.pdf]
