# Supplementary material for: A bone-specific adipogenesis pathway in fat-free mice defines key origins and adaptations of bone marrow adipocytes with age and disease
Source: eLife. 2021 Aug 11;10:e66275. doi: 10.7554/eLife.66275 (PMC8412938; doi:10.7554/eLife.66275)
Supplement: Supplementary file 1. [file elife-66275-supp1.docx]

**Supplementary File 1. Antibodies used for western blot and immunostaining.**

| Western Blot |  |  |  |  |
| --- | --- | --- | --- | --- |
| Primary Antibody  (Vendor, Cat. No) | Dilution | Secondary Antibody  (Vendor, Cat. No) | Conjugate | Dilution |
| Rabbit polyclonal Anti-Adiponectin, (Sigma-Aldrich, A6354, RRID:AB_1078104) | 1:1000 | Anti-Rabbit | HRP | 1:10,000 |
| Paraffin IHC |  |  |  |  |
| Primary Antibody  (Vendor, Cat. No) | Dilution | Secondary Antibody  (Vendor, Cat. No) | Conjugate | Dilution |
| Anti-CD68  (Abcam, UK, ab125212, RRID:AB_10975465) | 1:1000 | ImmPRESS Reagents (Vector Labs, MP-7401, RRID:AB_2336529) | HRP | N/A |
| Paraffin Immunofluorescence |  |  |  |  |
| Primary Antibody  (Vendor, Cat. No) | Dilution | Secondary Antibody  (Vendor, Cat. No) | Fluorophore | Dilution |
| Anti-Perilipin  (Progen Biotechnik, Germany, GP29, RRID:AB_2892611) | 1:400 | Donkey Anti-Guinea Pig  (Jackson IR, USA, 706-605-148, RRID:AB_2340476) | AF647 | 1:200 |
| Frozen Immunofluorescence |  |  |  |  |
| Primary Antibody (Vendor, Cat. No) | Dilution | Secondary Antibody | Fluorophore | Dilution |
| Anti-GFP  (Abcam, UK, ab13970, RRID:AB_300798) | 1:1000 | Donkey Anti-Chicken  (Jackson IR, USA, 703-545-155, RRID:AB_2340375) | AF488 | 1:500 |
| Anti-RFP  (Abcam, UK, ab62341, RRID:AB_945213) | 1:500 | Donkey Anti-Rabbit  (Jackson IR, USA, 711-585-152, RRID:AB_2340621) | AF594 | 1:500 |
| Anti-Perilipin  (Progen Biotechnik, Germany, GP29, RRID:AB_2892611)) | 1:500 | Donkey Anti-Guinea Pig  (Jackson IR, USA, 706-605-148, RRID:AB_2340476) | AF647 | 1:500 |
| ICC-IF |  |  |  |  |
| Primary Antibody (Vendor, Cat. No) | Dilution | Secondary Antibody | Fluorophore | Dilution |
| Anti-GFP  (Abcam, UK, ab13970, RRID:AB_300798) | 1:1000 | Donkey Anti-Chicken  (Jackson IR, USA, 703-545-155, RRID:AB_2340375) | AF488 | 1:500 |
| Anti-RFP  (Abcam, UK, ab62341, RRID:AB_945213) | 1:500 | Donkey Anti-Rabbit  (Jackson IR, USA, 711-585-152, RRID:AB_2340621) | AF594 | 1:500 |
| Anti-Perilipin  (Progen Biotechnik, Germany, GP29, RRID:AB_2892611)) | 1:500 | Donkey Anti-Guinea Pig  (Jackson IR, USA, 706-605-148, RRID:AB_2340476) | AF647 | 1:500 |
