# Supplementary material for: A bone-specific adipogenesis pathway in fat-free mice defines key origins and adaptations of bone marrow adipocytes with age and disease
Source: eLife. 2021 Aug 11;10:e66275. doi: 10.7554/eLife.66275 (PMC8412938; doi:10.7554/eLife.66275)
Supplement: Supplementary file 2. [file elife-66275-supp2.docx]

**Supplementary File 2. qPCR Primers**

| Gene | Primer sequence (Forward) | Primer sequence (Reverse) | Target Refseq |
| --- | --- | --- | --- |
| *Adipoq* | AAGAAGGACAAGGCCGTTCTCTT | GCTATGGGTAGTTGCAGTCAGTT | NM_009605 |
| *Ppia* | CACCGTGTTCTTCGACATCA | CAGTGCTCAGAGCTCGAAAGT | NM_008907 |
| *Tbp* | ACCTTATGCTCAGGGCTTGG | GCCGTAAGGCATCATTGGAC | NM_013684 |
| *Pparg* | GGAAAGACAACGGACAAATCAC | TACGGATCGAAACTGGCAC | NM_011146 |
| *Cebpa* | TGGACAAGAACAGCAACGAG | TCACTGGTCAACTCCAGCAC | NM_007678 |
| *Cxcl12* | TGCATCAGTGACGGTAAACCA | CACAGTTTGGAGTGTTGAGGAT | NM_001012477 |
| *Cfd* | ACATGGCTTCCGTGCAAGT | CAGAGTCGTCATCCGTCACTC | NM_013459.4 |
| *Retn* | CTAAGTCCTCTGCCACGTACC | GGCTGCTGTCCAGTCTATCC | NM_001204959.1 |
| *CD36* | CCTTGGCAACCAACCACAAA | CGCCAACTCCCAGGTACAAT | NM_001159555.1 |
| *Alpl* | ATAACGAGATGCCACCAGAGG | TTCCACATCAGTTCTGTTCTTCG | NM_007431 |
| *Adrb2* | TGGTTGGGCTACGTCAACTC | TCCGTTCTGCCGTTGCTATT | NM_007420 |
| *Adrb3* | CACCGCTCAACAGGTTTGATG | TCTTGGGGCAACCAGTCAAG | NM_013462 |
| *Pnpla2* | CAACGCCACTCACATCTACGG | GGACACCTCAATAATGTTGGCAC | NM_025802 |
| *Lipe* | CCATCAACCGACCAGGAGTG | CATGTTGGCCAGAGACGACAG | NM_001039507 |
| *Mgll* | TGCAGAGAGGCCAACCTACT | GTCAGCAGAACCCTCCGACT | NM_001166249 |
| *Dph1* | GCTGGTTGTGTCCGAGACTG | GCATTTGTAAGGCCACCTTCTT | NM_144491 |
| *Dph2* | GTGTAGTGGAGAGCCGGG | GAACTGCAAAGTCACCCGC | NM_026344.3 |
| *Dph3* | TGTGGGGATAACTTTGCCATCA | CTCCTTGTTGGTTGAAGGTGC | NM_001047433.2 |
| *Dnajc24 (Dph4)* | TGGTACAGCATTCTGGGTGC | TCTTAGCTCATCTTCATGCCGC | NM_026992 |
| *Dph5* | TTGGTGATCCATTTGGGGCT | CACACTCATGTACCGAGGGG | NM_027193.2 |
| *Dph6* | AAGTAGCAGCTTTGGGCTTAGAT | ATAGAGGGCAGTCCAACGTG | NM_001356438.1 |
| *Dph7* | GAGCACAACACGGCTAAACC | GCAGTCATCTCCCCCTGAATAC | NM_026044.4 |
